# Indications for Blood Transfusion and Exchange Transfusion in Sickle Cell Disease: A Single Center Experience

**DOI:** 10.7759/cureus.77499

**Published:** 2025-01-15

**Authors:** Essa Alsharari, Abdulelah Al Enazi, Ehab Hanafy, Mohammed Mustafa, Fadwa Abufara, Mustafa M Altoonisi

**Affiliations:** 1 Pediatric Department, King Salman Armed Forces Hospital, Tabuk, SAU; 2 Prince Sultan Oncology Center, King Salman Armed Forces Hospital, Tabuk, SAU

**Keywords:** acute chest syndrome, alloimmunization, exchange transfusion, hydroxyurea compliance, iron overload, pediatric transfusion, sickle cell anemia, simple transfusion, splenic sequestration, stroke prevention

## Abstract

Background

Sickle cell disease (SCD) is a prevalent inherited hemoglobinopathy characterized by chronic hemolytic anemia, vaso-occlusive crises (VOC), and multi-organ complications. Blood transfusion plays a critical role in the management of both acute and chronic complications associated with SCD, including stroke, acute chest syndrome (ACS), and splenic sequestration. However, the variability in transfusion practices and associated risks, such as alloimmunization and iron overload, necessitates further investigation into optimizing transfusion strategies in pediatric SCD patients.

Objectives

This study aimed to identify the most common indications for blood transfusion and exchange transfusion in pediatric patients with SCD at King Salman Armed Forces Hospital (KSAFH) in Tabuk, Saudi Arabia, from June 2020 to June 2024. By analyzing transfusion patterns and their outcomes, we sought to provide insights into improving transfusion management in this population.

Methods

An observational cross-sectional study was conducted on 99 pediatric SCD patients aged 1 to 14 years who received transfusions at KSAFH. Data were collected retrospectively from electronic medical records, including patient demographics, transfusion indications, hemoglobin levels, and adverse reactions. Descriptive statistical methods were used to assess the prevalence of various transfusion indications, and chi-square and analysis of variance (ANOVA) tests were applied to evaluate associations between clinical variables and transfusion outcomes.

Results

The study found that 68 patients (68.7%) required multiple transfusions, with exchange transfusions being used in 32 patients (31.3%), primarily for severe complications. ACS was the leading indication for transfusion, occurring in 32 patients (32.3%), followed by hemolytic crisis in 18 patients (18.2%), stroke prevention in 11 patients (11.1%), and splenic sequestration in eight patients (8.1%). Preoperative transfusions were administered to eight patients (8.1%), while seven patients (7.1%) required transfusions for aplastic crisis. More than 75% of ACS cases were managed with simple transfusions, and all showed significant improvement. Hydroxyurea non-compliance was notably high, with 64 patients (64.6%) not adhering to the regimen. Adverse transfusion reactions were rare, occurring in only four patients (4%), with fever being the most common reaction.

Conclusions

Blood transfusion remains an essential component of managing pediatric SCD, particularly for ACS, stroke prevention, and other severe complications. Simple transfusions were observed to be effective in many ACS cases, suggesting a role for less invasive interventions in specific clinical contexts. However, variability in transfusion practices and the high rate of hydroxyurea non-compliance highlight the need for standardized transfusion guidelines and enhanced patient education.

## Introduction

Sickle cell disease (SCD) is one of the most prevalent inherited hemoglobinopathies, characterized by chronic hemolytic anemia, vaso-occlusive crises (VOC), and multi-organ complications. The hallmark of the disease lies in the production of abnormal hemoglobin S (HbS), which results from a single point mutation in the β-globin gene. Under conditions of low oxygen tension, HbS polymerizes, causing red blood cells to assume a sickled shape. These sickled cells have reduced deformability, leading to vaso-occlusion and hemolysis, which in turn contributes to the chronic anemia, organ damage, and episodic crises seen in patients with SCD [1].

Blood transfusion has been a cornerstone of treatment for SCD, offering a critical intervention for both acute and chronic complications. Transfusion therapy aims to reduce the proportion of sickled erythrocytes in circulation, improving oxygen delivery and reducing the risk of VOC and other complications. However, despite its benefits, transfusion therapy comes with risks, including alloimmunization, iron overload, and transfusion reactions. The management of transfusion in SCD is further complicated by the variability in clinical presentation, which can range from mild to severe disease depending on factors such as genotype and environmental influences [2].

The indications for blood transfusion in SCD are diverse, spanning from acute conditions such as stroke, acute chest syndrome (ACS), and splenic sequestration to chronic management of severe anemia and stroke prevention. The decision to transfuse must balance the benefits of symptom relief and crisis prevention with the risks inherent in repeated blood transfusions [3]. In recent years, the approach to transfusion management has evolved, with a growing emphasis on exchange transfusion in specific high-risk scenarios such as ACS, stroke prevention, and perioperative care [4]. Exchange transfusion has the advantage of lowering the HbS percentage without significantly increasing the overall blood volume, thus reducing the risk of complications associated with simple transfusion [3,4].

The variability in transfusion practices highlights the need for clearer guidelines and a more standardized approach to managing transfusion in SCD. In Saudi Arabia, where the Arab Indian haplotype predominates, patients often exhibit higher levels of fetal hemoglobin (HbF), which has been shown to reduce the severity of clinical manifestations. Despite this protective effect, SCD remains a major public health concern in the region, and the management of transfusions continues to be a critical component of patient care [5].

Moreover, recent studies have highlighted the role of emerging therapeutic strategies, including transfusion support, in mitigating SCD-related complications. These approaches are particularly relevant in the context of chronic transfusion protocols and the prevention of complications such as stroke and ACS [6].

This study aims to address the gap in understanding the most common indications for blood transfusion and exchange transfusion in pediatric SCD patients at King Salman Armed Forces Hospital (KSAFH) in Tabuk, Saudi Arabia. Specifically, the objective is to identify the primary clinical scenarios necessitating transfusion interventions in this population, with a view to improving transfusion management practices and patient outcomes. By retrospectively analyzing data from 2020 to 2024, we seek to provide valuable insights into the patterns of transfusion use in this cohort.

## Materials and methods

Study design

This was an observational cross-sectional retrospective study conducted to determine the most common indications for blood transfusion and exchange in pediatric patients diagnosed with SCD at King Salman Armed Forces Hospital (KSAFH) in Tabuk, Saudi Arabia. The study focused on patients aged from birth to 14 years, receiving treatment from June 2020 till June 2024. The primary objective was to analyze the indications for blood transfusion and exchange, exploring both acute and chronic clinical scenarios requiring these interventions.

Data collection

The study involved a comprehensive review of medical records, capturing relevant clinical data through a customized data collection form. This form was tailored to extract crucial variables, including patient demographics (age, gender), clinical features (hemoglobin genotype), indications for blood transfusion or exchange, the frequency of transfusions, pre-transfusion hemoglobin levels, and any adverse reactions documented post-transfusion. The records were sourced from the hospital’s electronic medical record (EMR) system, covering transfusion logs, clinical notes, and laboratory data for each patient. Data collection extended to evaluating the primary reasons for blood transfusion and exchange, which included acute complications such as stroke and ACS, as well as chronic anemia management and preoperative preparation.

Inclusion criteria

Patients were included in the study based on specific criteria. Eligible participants were those diagnosed with SCD, confirmed through hemoglobin electrophoresis at KSAFH. The study focused on individuals aged from birth to 14 years at the time of receiving a blood transfusion or exchange. Inclusion required patients to have undergone at least one blood transfusion or exchange during the study period (2020-2024) and to have complete and relevant medical records available, including transfusion details and associated clinical notes.

Data extraction

Data extraction was performed systematically by trained medical personnel, ensuring accuracy and consistency. The data collection process involved retrieving information from multiple sources within the EMR, including laboratory results, transfusion reports, and clinical notes. Special attention was paid to identifying the indications for blood transfusion, which were classified into categories such as ACS, stroke prevention, hemolytic crisis, aplastic crisis, splenic sequestration, and other relevant clinical conditions. Each patient’s medical history was meticulously reviewed to ensure that the primary indication for blood transfusion was accurately documented. In cases where multiple transfusions or exchanges were performed, the frequency and timing were also recorded.

Data analysis

All data was collected and entered in SPSS version 26 (IBM SPSS Statistics for Windows, Version 26.0. Armonk, NY: IBM Corp). A descriptive analysis of the variables was presented and tabulated via frequencies, percentages, mean, standard deviations, and ranges. Furthermore, in inferential statistics, the Chi-Square or Fisher exact test is used to find the relationship between the categorical variables of the study. To find any difference in the lab results among blood transfusion and indications, analysis of variance (ANOVA) is used. A p-value less than or equal to 5 (p≤0.05) was considered statistically significant.

Ethical considerations

This study adhered to strict ethical guidelines, with all necessary approvals obtained from the Institutional Review Board (IRB) of King Salman Armed Forces Hospital (KSAFH) with approval number KSAFH-RET-2024-585. Patient confidentiality was maintained throughout the research process, with all personal identifiers removed from the dataset prior to analysis. Data were stored securely, ensuring compliance with institutional and international ethical standards for human research.

## Results

Descriptive findings

A total of 99 pediatric patients with SCD were included in this study. The patients' ages ranged from 1 to 14 years, with a mean age of 7.2±3.3 years. The average weight was 19.8±8.8 kg, and their pre-transfusion hemoglobin levels ranged between 4.8 and 9.6 g/dL, with a mean of 7.1±1.1 g/dL. The hematocrit (Hct) levels ranged from 14.2% to 28.5%, averaging 20.5±3.1%. White blood cell (WBC) counts varied from 2.1 to 23.6 x10^9^/L, with an average of 12.3±4.9 x10^9^/L. Platelet counts (Plt) had a broad range, from 21 to 705 x10^9^/L, with a mean of 285.4±175 x10^9^/L. Reticulocyte counts varied significantly, from 0.8% to 25.9%, with an average of 9.4±5.6%. The ferritin levels, reflecting iron stores, ranged from 57 to 3436 ng/mL, with a mean of 613.8±668.6 ng/mL. The mean corpuscular volume (MCV) ranged from 69.7 to 735, with an average of 88.4±66.0 fL (Table 1).

**Table 1:** Descriptive statistics of hematologic and demographic variables in pediatric SCA patients. Summary of the minimum, maximum, mean, and standard deviation values for key variables including age, weight, hemoglobin, hematocrit, white blood cell count (WBC), platelet count, reticulocyte count (retics), ferritin levels, and mean corpuscular volume (MCV) in pediatric sickle cell anemia patients.

Variables	Minimum	Maximum	Mean	Standard deviation
Age (years)	1	14	7.2	3.3
Weight (Kg)	8.0	51.0	19.8	8.8
Hemoglobin (g/dL)	4.8	9.6	7.1	1.1
Hematocrit (%)	14.2	28.5	20.5	3.1
WBC (x10^9^/L)	2.1	23.6	12.3	4.9
Platelets (x10^9^/L)	21.0	705.0	285.4	175.0
Retics (%)	0.8	25.9	9.4	5.6
Ferritin (ng/mL)	57.0	3436.0	613.8	668.6
MCV	69.7	735.0	88.4	66.0

Patient characteristics

The study population predominantly consisted of males (60, 60.6%), with females accounting for 39 (39.4%). The age distribution showed that the largest group of patients, 42 (42.4%), were between 6 and 10 years old, followed by 35 (35.4%) of patients between 1 and 5 years old, and 22 (22.2%) aged 11 to 14 years (Table 2).

**Table 2:** Distribution of demographic, clinical variables, and treatment compliance in pediatric sickle cell anemia patients. This table presents the frequency and percentage distribution of key variables, including age, gender, number of transfusions and exchanges, type of sickle cell disease, comorbidities, clinical indications for transfusion, adverse reactions, additional transfusion needs, and hydroxyurea compliance, in pediatric patients with sickle cell anemia.

Variables		Frequency	Percent
Age	1-5 Years	35	35.4
	6-10 Years	42	42.4
	11-14 Years	22	22.2
Gender	Male	60	60.6
	Female	39	39.4
Number of transfusions	1 time	2	2.0
	2 times	4	4.0
	3 times	2	2.0
	4 times	8	8.1
	5 times	11	11.1
	6 times	4	4.0
	>6 times	68	68.7
Number of exchanges	None	68	68.7
	1 time	14	14.1
	2 times	5	5.1
	3 times	3	3.0
	4 times	2	2.0
	5 times	3	3.0
	>5 times	4	4.0
Type of sickle cell disease	HbSS	95	96.0
	HBS beta thalassemia	4	4.0
Comorbidities	None	38	39.5
	Asthma	56	58.3
	Congenital heart disease	1	1
	Joubert syndrome	1	1
Indications	Acute chest syndrome	32	32.3
	Acute pain crisis	3	3
	Acute pain crisis, severe anemia	4	4
	Aplastic crisis	7	7.1
	Hemolytic crisis	18	18.2
	Hepatic sequestration	1	1
	Hypersplenism	1	1
	Preoperative management	8	8.1
	Sepsis	6	6.1
	Splenic sequestration	8	8.1
	Stroke prevention	11	11.1
Adverse reactions	None	95	96
	Fever	2	2
	Hemolysis	1	1
	Allergy	1	1
Need of additional transfusion/exchange	Yes	49	49.5
	No	50	50.5
Hydroxyurea compliance	Compliance	35	35.4
	Non-compliance	64	64.6

Transfusion and exchange frequency

Approximately 70% of the patients (68, 68.7%) required more than six blood transfusions during the study period, while only 2% (2 patients) had undergone a single transfusion. A total of 31% of patients (32, 31.3%) required at least one exchange transfusion, with 14% (14 patients) having only one exchange. The majority of patients (68, 68.7%) did not require an exchange transfusion (Table 2).

Clinical indications for transfusion/exchange

ACS was the leading indication for transfusion, occurring in 32 patients (32.3%), followed by hemolytic crisis in 18 patients (18.2%), stroke prevention in 11 patients (11.1%), and splenic sequestration in 8 patients (8.1%). Other indications included aplastic crisis in 7 patients (7.1%), preoperative management in eight patients (8.1%), and sepsis in six patients (6.1%). Acute pain crisis was a rare indication for transfusion, occurring in only three patients (3%) (Table 2).

Comorbidities and complications

Asthma was the most prevalent comorbidity, affecting 56 patients (58.3%), while 39 patients (39.5%) had no comorbidities. Congenital heart disease and Joubert syndrome were observed in one patient each (1% each). Adverse reactions to transfusions were rare, with 95 patients (96%) experiencing no complications. The most common adverse reaction was fever, seen in two patients (2%), followed by hemolysis and allergic reactions, each occurring in one patient (1%) (Table 2).

Hydroxyurea compliance

Non-compliance with hydroxyurea therapy was notably high, with 64 patients (64.6%) not adhering to the prescribed regimen. The compliance rate was only 35 patients (35.4%). Interestingly, hydroxyurea non-compliance was particularly high in patients with ACS, where non-compliant patients outnumbered compliant ones by more than double (Table 2).

Statistical analysis

A Chi-square test was performed to evaluate the association between the number of transfusions and patient demographics (age, gender, and sickle cell genotype). No statistically significant association was found between transfusion frequency and age, gender, or genotype (p>0.05). Similarly, no significant association was observed between the type of indication and the number of transfusions or exchange transfusions (p>0.05) (Table 3).

**Table 3:** Association between patient demographics, sickle cell disease type, and number of transfusions in pediatric SCD patients. This table illustrates the distribution of transfusion frequency by age group, gender, and type of sickle cell disease (SCD) in pediatric patients; either hemoglobin SS (HbSS) or hemoglobin S beta thalassemia, along with the corresponding chi-square and p-values for statistical significance.

Variables		Number of transfusions							Chi-square/Fisher Exact	p-value
		1 time	2 times	3 times	4 times	5 times	6 times	>6 times		
Age in years	1-5	1	3	1	5	5	1	19	16.25	0.071
	6-10	0	0	0	2	6	3	31		
	11-14	1	1	1	1	0	0	18		
Gender	Male	1	2	1	7	6	1	42	5.65	0.463
	Female	1	2	1	1	5	3	26		
Type of SCD	HbSS	1	3	2	8	11	4	66	10.87	0.094
	HBS beta thalassemia	1	1	0	0	0	0	2		

Laboratory findings (ANOVA results)

An ANOVA was applied to examine the differences in laboratory findings among patients with varying numbers of transfusions and different clinical indications. The ANOVA test did not show any statistically significant differences in laboratory values (hemoglobin, hematocrit, WBC, platelet count, reticulocyte count, ferritin, and MCV) across the number of transfusions (p > 0.05). However, significant differences were noted when analyzing the indications for transfusion. Hemoglobin and hematocrit levels were significantly higher in patients undergoing transfusions for preoperative management (p=0.001). WBC counts were markedly elevated in patients with hypersplenism (p=0.014), while platelet counts were significantly lower in both hypersplenism and splenic sequestration cases (p=0.032). Additionally, reticulocyte counts were significantly reduced in patients experiencing aplastic crisis (p=0.034) (Table 4).

**Table 4:** Laboratory parameters of clinical indications for transfusion in pediatric SCA patients. This table presents the mean, standard deviation (SD), F-statistics, and p-values for laboratory parameters (hemoglobin (Hb), hematocrit (Hct), white blood cell count (WBC), platelet count (Plt), reticulocytes (retics), ferritin levels, and mean corpuscular volume (MCV)) across different clinical indications, such as acute chest syndrome, splenic sequestration, and stroke prevention, in pediatric sickle cell anemia patients.

Lab works	Indications	Mean	SD	F-statistics	p-value
Hb (g/dL)	Acute chest syndrome	6.9	0.9	4.19	0.001
	Acute pain crisis	7.1	0.8		
	Acute pain crisis, severe anemia	6.7	0.3		
	Aplastic crisis	6.5	0.8		
	Hemolytic crisis	6.7	0.7		
	Hepatic sequestration	6.6	0.0		
	Hypersplenism	6.8	0.0		
	Preoperative management	8.8	0.5		
	Sepsis	7.4	0.9		
	Splenic sequestration	6.9	0.7		
	Stroke prevention	7.7	1.7		
Hct (%)	Acute chest syndrome	19.5	3.0	3.22	0.001
	Acute pain crisis	20.0	0.9		
	Acute pain crisis, severe anemia	20.2	1.5		
	Aplastic crisis	19.7	2.3		
	Hemolytic crisis	19.6	2.3		
	Hepatic sequestration	18.4	0.0		
	Hypersplenism	19.2	0.0		
	Preoperative management	24.9	1.3		
	Sepsis	21.4	2.1		
	Splenic sequestration	20.8	2.3		
	Stroke prevention	22.3	4.5		
WBC (x10^9^/L)	Acute chest syndrome	12.2	4.9	2.42	0.014
	Acute pain crisis	12.0	5.9		
	Acute pain crisis, severe anemia	17.5	4.2		
	Aplastic crisis	8.5	4.8		
	Hemolytic crisis	13.2	3.8		
	Hepatic sequestration	14.6	0.0		
	Hypersplenism	23.4	0.0		
	Preoperative management	12.0	3.6		
	Sepsis	15.4	5.5		
	Splenic sequestration	11.6	3.6		
	Stroke prevention	9.4	5.2		
Plt (x10^9^/L)	Acute chest syndrome	291.6	151.6	2.11	0.032
	Acute pain crisis	309.3	112.9		
	Acute pain crisis, severe anemia	442.0	210.3		
	Aplastic crisis	143.6	110.8		
	Hemolytic crisis	316.4	212.5		
	Hepatic sequestration	449.0	0.0		
	Hypersplenism	67.0	0.0		
	Preoperative management	354.0	184.7		
	Sepsis	316.5	128.6		
	Splenic sequestration	126.5	48.5		
	Stroke prevention	297.0	192.5		
Retics (%)	Acute chest syndrome	7.2	4.0	2.10	0.034
	Acute pain crisis	13.5	7.4		
	Acute pain crisis, severe anemia	8.5	5.3		
	Aplastic crisis	4.5	2.5		
	Hemolytic crisis	11.3	5.1		
	Hepatic sequestration	16.8	0.0		
	Hypersplenism	12.5	0.0		
	Preoperative management	9.4	6.0		
	Sepsis	10.4	6.2		
	Splenic sequestration	11.7	6.1		
	Stroke prevention	11.7	7.4		
Ferritin(ng/mL)	Acute chest syndrome	1291.3	1453.0	0.71	0.680
	Acute pain crisis	57.0	0.0		
	Acute pain crisis, severe anemia	387.0	0.0		
	Aplastic crisis	0.0	0.0		
	Hemolytic crisis	530.8	416.7		
	Hepatic sequestration	0.0	0.0		
	Hypersplenism	605.0	0.0		
	Preoperative management	717.3	603.3		
	Sepsis	360.5	429.2		
	Splenic sequestration	255.7	286.0		
	Stroke prevention	589.0	333.5		
MCV	Acute chest syndrome	80.8	7.0	1.38	0.202
	Acute pain crisis	84.4	10.8		
	Acute pain crisis, severe anemia	91.1	7.4		
	Aplastic crisis	82.7	3.2		
	Hemolytic crisis	82.4	6.9		
	Hepatic sequestration	85.4	0.0		
	Hypersplenism	71.6	0.0		
	Preoperative management	81.7	6.7		
	Sepsis	83.1	6.3		
	Splenic sequestration	79.2	5.8		
	Stroke prevention	79.8	7.0		

Need for additional transfusions

Approximately 50% of the patients required additional transfusions or exchanges. This need was highest among patients with ACS, hemolytic crisis, and those undergoing stroke prevention, but no statistically significant difference was observed in the frequency of additional transfusions based on clinical indications (p>0.05) (Table 5).

**Table 5:** Relationship between transfusion/exchange frequency and clinical indications in pediatric SCA patients. This table presents the distribution of transfusion and exchange frequencies based on clinical indications such as acute chest syndrome, splenic sequestration, and stroke prevention in pediatric sickle cell anemia patients, along with the corresponding Chi-square (χ²) test values, degrees of freedom (df), and p-values for statistical analysis.

Variables		Indications											Chi-square/ Fisher Exact (df)	p-value
		Acute chest syndrome	Acute pain crisis	Acute pain crisis, severe anemia	Aplastic crisis	Hemolytic crisis	Hepatic sequestration	Hypersplenism	Preoperative management	Sepsis	Splenic sequestration	Stroke prevention		
Transfusions	1 time	0	0	0	1	0	0	0	1	0	0	0	χ2=75.45, df=54	0.086
	2 times	0	0	0	1	2	1	0	0	0	0	0		
	3 times	0	0	0	0	1	0	0	0	1	0	0		
	4 times	3	0	1	1	1	0	0	1	0	1	0		
	5 times	3	0	2	1	3	0	0	1	1	0	0		
	6 times	0	0	0	1	1	0	0	0	0	1	1		
	>6 times	26	3	1	2	10	0	1	5	4	6	10		
Exchanges	None	20	2	4	6	16	1	1	5	5	8	0	χ2=65.81, df=45	0.283
	1 time	6	1	0	1	1	0	0	1	1	0	3		
	2 times	3	0	0	0	0	0	0	1	0	0	1		
	3 times	1	0	0	0	1	0	0	0	0	0	1		
	4 times	1	0	0	0	0	0	0	0	0	0	1		
	5 times	1	0	0	0	0	0	0	1	0	0	1		
	>5 times	0	0	0	0	0	0	0	0	0	0	4		
Additional transfusion/exchange	Yes	17	1	1	2	9	1	1	2	2	6	7	χ2=10.22, df=9	0.421
	No	15	2	3	5	9	0	0	6	4	2	4		

Age, gender, and indications

A Chi-square test revealed a statistically significant association between gender and the type of clinical indication (p=0.04). Male patients were more likely to present with ACS and hemolytic crisis, while splenic sequestration was more commonly seen in females. However, no statistically significant relationship was found between age and the type of clinical indication (p>0.05) (Table 6).

**Table 6:** Relationship between clinical indications, age, gender, and hydroxyurea compliance in pediatric SCA patients. This table shows the distribution of clinical indications for transfusion by age group, gender, and hydroxyurea compliance in pediatric sickle cell disease.

Indications	Age in years			Fisher Exact	p-value	Gender		Fisher Exact	p-value	Hydroxyurea		Fisher Exact	p-value
	1-5	6-10	11-14			Male	Female			Compliance	Non-Compliance		
Acute chest syndrome	11	11	10	22.6	0.20	18	14	16.8	0.04	9	23	12.2	0.22
Acute pain crisis	1	1	1			3	0			2	1		
Acute pain crisis, severe anemia	1	3	0			4	0			3	1		
Aplastic crisis	3	4	0			4	3			3	4		
Hemolytic crisis	7	6	5			12	6			8	10		
Hepatic sequestration	1	0	0			0	1			1	0		
Hypersplenism	1	0	0			0	1			0	1		
Preoperative management	1	3	4			5	3			3	5		
Sepsis	3	3	0			4	2			3	3		
Splenic sequestration	5	3	0			1	7			2	6		
Stroke prevention	1	8	2			9	2			1	10		

## Discussion

SCD is a devastating inherited hemoglobinopathy with far-reaching clinical and socio-economic implications. Despite advancements in treatment, transfusion therapy remains a cornerstone of managing both acute and chronic complications of SCD, particularly in pediatric populations. The results of this study, focusing on pediatric SCD patients treated at KSAFH from June 2020 to June 2024, offer valuable data about transfusion patterns and outcomes in this specific context.

The role of blood transfusion in SCD

Blood transfusions, both simple and exchange, are critical for alleviating the complications associated with SCD. The primary objective of transfusion in SCD is to reduce the proportion of sickled erythrocytes in circulation, thereby improving oxygen delivery and mitigating the risk of VOC and other complications, such as ACS and stroke [7,8]. This approach was well-reflected in our cohort, where approximately 70% of patients required multiple transfusions, and exchange transfusions were performed in 31% of cases, mostly for severe complications.

The use of transfusion for stroke prevention has been well documented in previous literature, with strong evidence supporting its efficacy in reducing both primary and secondary stroke risks [9,10]. This was consistent with the findings in our study, where 11.1% of patients received transfusions specifically for stroke prevention. Howard et al. highlighted the benefits of chronic transfusions in pediatric SCD patients to prevent strokes, reinforcing the value of regular monitoring and proactive management [10]. In our cohort, most patients undergoing transfusion in acute settings were treated with exchange transfusions, while simple transfusions were primarily reserved for cases on a prophylactic regimen with low hemoglobin, allowing for safe transfusion. Additionally, we ensured that the proportion of HbS was maintained around 30%, as recommended, to optimize safety and efficacy in preventing stroke and other complications.

Moreover, transfusion plays a critical role in managing ACS, the most common cause of transfusion in our cohort, affecting 32.3% of patients. This is in line with global trends that show ACS as a leading cause of morbidity in SCA [8,11]. The pathophysiology of ACS involves vaso-occlusion in the pulmonary microvasculature, leading to hypoxia, inflammation, and acute lung injury [12,13]. Timely transfusion can rapidly improve oxygenation by decreasing the proportion of sickled cells, and exchange transfusions are particularly beneficial for severe cases of ACS [14]. In our cohort, more than 75% of the patients with ACS underwent simple transfusions rather than exchange transfusions, and all showed significant improvement. This suggests that for many ACS cases, simple transfusion can be effective in managing the condition, potentially reducing the need for more complex procedures like exchange transfusion in less severe instances.

Splenic sequestration is another significant complication in SCD, characterized by the sudden pooling of blood in the spleen, which can lead to acute anemia, hypovolemic shock, and potentially death if not treated promptly. In our cohort, splenic sequestration accounted for 8.1% of transfusion cases. This complication is more common in younger patients, as the spleen often becomes infarcted and non-functional in older children with SCD [12]. Simple transfusion was the primary intervention for these patients, aimed at increasing hemoglobin levels and stabilizing hemodynamics. Studies, including those by Wallace et al. [13], suggest that early intervention with transfusion can prevent further deterioration and improve outcomes, reducing the need for more invasive procedures like splenectomy. However, our approach is to maintain the patient on transfusions until performing splenectomy, ideally at or after the age of three, when the general condition and circumstances allow, especially in cases of major splenic sequestration.

Additionally, preoperative transfusion is a well-established strategy for reducing the risk of complications in patients with SCD undergoing surgery. In our study, 8.1% of patients required transfusions for preoperative preparation. The goal of transfusion in these cases is to raise hemoglobin levels and reduce the proportion of sickled cells, minimizing the risk of perioperative vaso-occlusive events and other complications [15]. Most of our patients received simple transfusions prior to surgery, in accordance with the recommendations to maintain HbS levels below 30%, which has been shown to improve surgical outcomes and reduce post-operative complications [16].

One of the most life-threatening complications in SCD is aplastic crisis, often triggered by infections, particularly parvovirus B19, which suppresses bone marrow function. In our cohort, 7.1% of patients required transfusions due to aplastic crisis. This is consistent with other studies that describe aplastic crisis as a relatively rare but severe complication requiring urgent intervention [12]. Simple transfusion was the preferred approach in these cases, as it provides immediate relief by replenishing the depleted red cell mass and stabilizing the patient. Ziemba et al. [14] have demonstrated the efficacy of transfusion in managing aplastic crisis, emphasizing the need for prompt recognition and treatment to prevent severe anemia and further complications.

The evolution of transfusion strategies​​​​​​ (simple vs exchange transfusion)**

The decision between simple transfusion and exchange transfusion is influenced by several factors, including the severity of the clinical scenario, the patient’s baseline hemoglobin levels, and the need to avoid complications such as iron overload. Simple transfusions increase the total hemoglobin concentration but can lead to iron overload and hyperviscosity, particularly when administered frequently [8,17]. In contrast, exchange transfusion removes sickled erythrocytes while minimizing iron accumulation, making it the preferred choice for chronic management and acute complications like ACS and stroke [10,18,19].

Our study found that exchange transfusion was used in 31% of patients, primarily for stroke prevention and severe ACS. This finding is supported by Ziemba et al., who demonstrated that automated red cell exchange offers significant advantages in maintaining target HbS levels without causing excessive iron accumulation [14]. The use of automated exchange also reduces the frequency of transfusion-related complications, such as alloimmunization, which is a major concern in patients undergoing repeated transfusions [13]. The advantages of exchange transfusion are further supported by studies like those of Merlin et al. and Hequet et al., which discuss the efficacy of red blood cell exchange in both emergency and chronic settings [8,9]. In particular, the studies emphasize that exchange transfusions can rapidly reduce HbS levels and stabilize patients with severe VOC or those at risk of stroke [7,14]. However, they also highlight the need for careful monitoring to prevent complications like hypovolemia, especially in pediatric patients [9].

Regional variability in SCD presentation and transfusion practices

The Arab-Indian haplotype of SCD, which predominates in Saudi Arabia, is characterized by higher levels of HbF, offering a protective effect against some of the more severe manifestations of SCD [11,13]. Despite this protective effect, SCD remains a significant public health issue in the region, with transfusion therapy being a key component of managing the disease. In this study, the majority of patients required multiple transfusions, with ACS and hemolytic crises being the most common indications [13,14]. These findings align with other studies conducted in the region, where the high prevalence of SCD necessitates frequent transfusion interventions [11,14]. The variability in clinical presentation, ranging from mild to severe disease, underscores the importance of individualized treatment plans tailored to each patient’s needs [9,13].

Risks and complications of transfusion therapy

Despite its therapeutic benefits, transfusion therapy is not without risks. Alloimmunization, iron overload, and transfusion reactions are well-documented complications that can significantly impact patient outcomes [8,17,18]. In our cohort, adverse transfusion reactions were rare, occurring in only 4% of patients. This included febrile reactions, hemolysis, and allergic responses, consistent with other studies that report similar rates of complications [13,14].

Alloimmunization, in particular, is a significant concern in patients requiring chronic transfusion, as the development of antibodies can limit the availability of compatible blood and increase the risk of hemolytic reactions [11,18]. Strategies to mitigate this risk include extended antigen matching and the use of automated red cell exchange, which reduces the overall exposure to donor red cells [8,10]. In our study, iron overload was managed effectively in most patients, with regular monitoring of ferritin levels and the use of iron chelation therapy in those with significant iron accumulation.

Howard et al. discussed the importance of regular monitoring for iron overload and emphasized the need for individualized iron chelation protocols to prevent long-term complications such as liver and heart disease [10]. In our cohort, the average ferritin level was 613.8 ng/mL, which is within the expected range for patients undergoing chronic transfusion therapy [13]. However, the wide range of ferritin values observed (57 to 3436 ng/mL) highlights the variability in iron burden among patients and underscores the need for personalized management plans [11].

Hydroxyurea non-compliance and its clinical implications

Hydroxyurea is a cornerstone of SCD management, significantly reducing the frequency of VOC and the need for transfusions by inducing HbF production [13,18]. However, non-compliance with hydroxyurea therapy was notably high in our cohort, with 64.6% of patients not adhering to the prescribed regimen. This non-compliance was particularly evident among patients with ACS, where non-compliant patients outnumbered compliant ones by more than twofold.

The high rate of non-compliance with hydroxyurea therapy is concerning, as it directly correlates with an increased need for transfusions and a higher risk of severe complications [7,11]. Previous studies have shown that hydroxyurea can significantly reduce the need for transfusion by up to 50% in compliant patients [18]. Additionally, in a systematic review by Pandey et al., even low-dose hydroxyurea (10 mg/kg/day) was found to significantly reduce the frequency of VOC, hospitalizations, and the need for blood transfusions in SCD patients. HbF levels were reported in 80% of the studies (13 studies), all of which demonstrated an increase. Across these studies, the mean HbF levels rose from 15.8% to 21.4% [20]. Furthermore, current best practices support long-term hydroxyurea therapy at the maximum tolerated dose (MTD), which is well-tolerated by pediatric patients with SCD and demonstrates sustained hematologic efficacy with apparent long-term safety [21]. This finding underscores the importance of patient education and adherence monitoring, particularly in regions where the burden of SCD is high [8].

The need for standardized transfusion guidelines

One of the key findings of this study is the variability in transfusion practices, particularly concerning the decision to use simple versus exchange transfusion. While exchange transfusion offers clear benefits in specific clinical scenarios, such as stroke prevention and ACS, there remains a lack of standardized guidelines to dictate when and how these interventions should be implemented [9,18].

The variability in transfusion practices observed in this study mirrors the findings of Estcourt et al., who highlighted the need for clearer transfusion guidelines in managing SCD [19]. In their review, they found significant differences in transfusion protocols across different regions and institutions, which could impact patient outcomes [19]. Standardized guidelines tailored to the specific needs of pediatric SCD patients are crucial for optimizing transfusion therapy and minimizing complications such as iron overload and alloimmunization [11,13]. Incorporating advanced AI technologies could further enhance these guidelines by integrating large-scale patient data and updated clinical evidence into personalized patient management, as demonstrated in recent studies on AI-driven healthcare advancements [22].

Limitations of the study

One of the main limitations of this study is its retrospective design, which inherently relies on the accuracy and completeness of medical records. This dependence on pre-existing data, particularly from EMRs, can result in missing or incomplete information, potentially affecting the robustness of the findings. Additionally, this study was conducted at a single center, KSAFH, which may limit the generalizability of the results to other regions or healthcare settings, especially in areas with different SCD genotypes or management practices. Another limitation is the relatively small sample size of 99 pediatric patients, which may not capture the full spectrum of disease severity and treatment responses seen in larger populations. Lastly, while this study focused on transfusion practices and their outcomes, other therapeutic modalities, such as hydroxyurea compliance, were not explored in depth, potentially overlooking other influential factors in managing SCD.

## Conclusions

This study highlights the critical role of transfusion therapy in the management of pediatric SCD, particularly in cases of ACS, stroke prevention, and severe complications like splenic sequestration and aplastic crisis. Simple transfusions were effective in most ACS cases, while exchange transfusions were reserved for high-risk situations, such as stroke prevention. The findings also emphasize the need for standardized transfusion guidelines, as variability in transfusion practices was observed. Furthermore, the high rate of hydroxyurea non-compliance suggests the importance of improving patient education and adherence to this therapy for better long-term outcomes in managing SCD.
